# Synthesis, biological activity and molecular modeling study of novel 1,2,4-triazolo[4,3-*b*][1,2,4,5]tetrazines and 1,2,4-triazolo[4,3-*b*][1,2,4]triazines

**DOI:** 10.1038/s41598-020-62977-x

**Published:** 2020-04-09

**Authors:** Ahmed A. M. El-Reedy, N. K. Soliman

**Affiliations:** Basic Science Department, Faculty of Oral and Dental Medicine, Nahda University, Beni-Suef, Egypt

**Keywords:** Drug discovery and development, Molecular biology

## Abstract

Different novel 1,2,4-triazolo[4,3-b][1,2,4,5]tetrazines and 1,2,4-triazolo[4,3-b][1,2,4]triazines have been obtained from heterocyclization of 3-substituted-4-amino-5-substituted-amino-1,2,4-triazoles (**3a-d**) and 3-substituted-4-amino-5-hydrazino-1,2,4-triazoles (**9a,b)** with (α and β) bifunctional compounds like chloromethyl biphenyl-phosphanoxide, pyruvic acid, phenacyl bromide, diethyl oxalate, triethyl orthoformate, triethyl phosphite, fluorinated benzaldehydes, carbon disulfide and ethyl chloroformate under different experimental settings. Fourier transformer infrared analysis (FTIR), Proton nuclear magnetic resonance (^1^H NMR) and ^13^C nuclear magnetic resonance (^13^C NMR), as well as that of the mass spectral data, were used as the appropriate characterization techniques for the chemical structures of all newly synthesized compounds. The newly prepared compounds were examined as an anti-inflammatory, antibacterial agents (against *E. coli* (*Escherichia coli*) and *P. aeruginosa* (*Pseudomonas aeruginosa*) as examples for Gram-negative bacteria and *S. aureus* (*Staphylococcus aureus*) as examples for Gram-positive bacteria), as well as antifungal (against *C. albicans* (*Candida albicans*)) agents. The newly prepared compound showed high antibacterial, antifungal, and anti-inflammatory activities in comparing with the commercial antibiotics Indomethacin, Nalidixic acid, Imipenem, and Nystatin. Docking of the most active compounds was performed depending on the results of antibacterial screening and the anti-inflammatory assay.

## Introduction

Studies on heterocyclic compounds containing bridgehead nitrogen atom particularly those holding (1,2,4,5)-tetrazine, (1,2,4)-triazole and (1,2,4)-triazine derivatives have received much interest recently as they can be used in a variety of applications, especially in the medicinal field. For example, many of 1,2,4-triazole rings are found into a wide range of pharmaceutical drugs including antimicrobial agents^1,2^, antifungal^3,4^, antibacterial^5–9^, antimycobacterial^10^, antiviral^11,12^, anticancer^13^, antitubercular^14^, antimycotic activity^15,16^, antimigraini agents, anti-inflammatory and analgesic^17–19^, anticonvulsants^20^, antinociceptive^21^, anti-ureaese^22^, antioxidant^23^, CNS stimulants, and antidepressant^24^ properties.

1,2,4-triazole rings possess not only diverse pharmacological activities but also to have herbicidal, insecticidal, plant growth regulatory and antifungal activities^25^. Thus, for many years, the biochemistry of these molecules has been investigated^26^. Also, Heterobicyclic nitrogen systems containing 1,2,4-Triazines derivatives and 1,2,4-Triazines themselves have been found to display a diversity of biological applications such as Lamotrigine as anti-epileptic drug^27^, Tirapazamine as anti-tumor^28^, and fused 1,2,4–triazines as antimicrobial^29,30^, anti–viral^31^, antimycobacterial^32^, anxiolytic^33^ and antidepressant^34^ activities. They also have shown anti–HIV and anticancer activities^35^. Moreover, derivatives of the tetrazine ring have attracted extensive attention from numerous research groups because of their interesting and diverse biological activities as antitumor and antiviral^36^. In this respect, Abdel-Rahman *et al*.^37–39^ found that 1,2,4-triazines, 1,2,4-triazoles and/or 1,2,4-triazolo-1,2,4,5-tetrazines can be used as a molluscicidal and antimicrobial as well as they have anticancer drugs activity. Due to their novel energetic properties^25,26,40^, organic compounds with high-nitrogen content currently attract the attention of many researchers.

In light of these remarks, researchers were prompted to design and synthesize new drugs containing heterocyclic compounds, especially, those containing triazoles, triazines and tetrazines rings as a result of the fact that Nitrogen–Nitrogen bond is difficult to be produced in living organisms in addition to its therapeutic activities. Thus, because of these remarks, the target of this work is to synthesize some new 1,2,4-triazole fused with 1,2,4-triazine and/or 1,2,4,5-tetrazine nucleus holding different types of functional groups to enhance their biological activity, in a hope to design a semi-drug. To demonstrate whether COX-II was a potential target for our newly prepared compounds, molecular modeling studies have been conducted on these compounds.

## Experimental

### General Method for the preparation of novel 1,2,4-triazolo[4,3-b][1,2,4,5]tetrazines and 1,2,4-triazolo[4,3-b][1,2,4]triazines

A mixture of compounds **1a,b**, and **2a,b** with 1:1 molar ratio in 100 mL Ethanol-DMF was refluxed for 5 h, then poured into ice and the formed solids were collected by filtration and crystallized to give compounds **3a to 3d**, respectively. To prepare the compounds **5a–d**, a mixture of (0.01 mol) of **3a–d** and (0.01 mol) of chloromethyl-diphenylphosphanoxide in (100 mL) THF with (0.5 mL) TEA was refluxed for 5 h and cooled. The obtained solids were then were collected by filtration and crystallized to give **5a–d**, respectively. When (0.01 mol) of **3a–d** were mixed with (0.01 mol) of phenacyl bromide in 50 mL from 5% ethanolic KOH and heated under reflux for 3 h, then poured into ice and acidified with few drops of HCl. The yielded solids were filtered and crystallized to give compounds **6a–d** respectively. Compounds **7a–d** were prepared by mixing a mixture of (0.01 mol) **3a–d** and (0.01 mol) sodium pyruvate, in 10 mL H_2_O with 50 mL 5% aqueous NaOH. After that, the mixture was refluxed for 3 h, then poured into ice and acidified with few drops of HCl. The products filtered and recrystallized to give **7a–d compounds** respectively. Equimolar ratio mixture of both compounds **3a–d** and diethyl oxalate in 100 mL THF was refluxed for 5 h and cooled. The obtained products were filtered and crystallized from the proper solvent to give **8a–d** respectively. A mixture of each of **1a,b** (0.01 mol) and hydrazine hydrate (0.04 mol) in ethanol (100 mL) was refluxed for 6 h and cooled. Then a few drops of acetic acid were added. The produced solids were filtered off and recrystallized to give **9a,b** respectively^41,42^. When the equimolar ratio of each of **9a,b** and triethylphosphite in tetrahydrofuran (100 mL) and triethylamine (0.5 mL) was refluxed 6 h and cooled. The obtained solids were filtered off and recrystallized to give compounds **10a,b** respectively. A mixture of **9a,b** (0.01 mol) and triethylorthoformate (0.012 mol) in tetrahydrofuran (100 mL) was refluxed for 6 h and cooled. The produced solids were filtered off and recrystallized to give **11a,b** respectively. The spectral data together with the physical constants of **11a,b** are listed below. A mixture of **9a,b** (0.01mole) and 2-chloro-6-fluorobenzeldehyde (0.01 mol) in ethanol (100 mL)/piperidine (0.5 mL) was refluxed 12 h and then cooled. The produced solids were filtered off and crystallized to give **12a,b** respectively. A mixture of each of **9a,b** (0.01 mol), CS_2_ (0.02 mol) in DMF (50 mL) was refluxed for 3 h and cooled, then poured onto the ice. The resulting solids so formed were filtered off and recrystallized to give **13a,b** respectively. A mixture of each of **9a,b** (0.01 mol) and ethyl chloroformate (0.012 mol) with tetrahydrofuran (100 mL) and triethylamine (0.5 mL, added dropwise) was refluxed for 3 h and then cooled. The solids obtained were filtered off and recrystallized to give **14a,b** respectively.

The first compound (**1)** was prepared by direct hydrazinolysis of 4-pyridyl-CONHNHCS_2_K according to Reid *et al*. reported method^43^.

### Characterization of the newly synthesized samples

The spectral data together with the physical constants of all of the previously prepared compounds were identified as follows:

To confirm the occurrence of the reaction, Fourier transformer infrared (FTIR) spectrometer (a Perkin Lemer Spectrum RXI FT-IR systems No. 55529) was used. FT-IR spectra within the wavenumber ranged from 4000–600 cm^−1^ were recorded at room temperature in ATR discs.

^1^H/ ^13^C-NMR Spectra were recorded in (DMSO-*d*6) at 400 MHz with a Bruker NMR Advance DPX 400 MH Spectrometer. TMS was used as an internal reference. Chemical shifts were supposed to be due to the presence of the solvent.

The melting points of the synthesized compounds were determined by using SMP (Stuart Scientific melting point) (USA).

GCMS-Q 1000 Ex spectrometer was used to measure the mass spectra of the prepared materials. Shimadzu UV-visible 3101 PC Spectrophotometer was used to record the electronic absorption spectra of the synthesized materials in DMF.

#### Antimicrobial activity

A bacterial suspension having a density of about 1 to 2 ×10^8^ (CFU) colony-forming units/ mL was prepared by aging a single colony on an agar plate for 24 hours. The antibacterial activity of some newly synthesized compounds was investigated on Mueller-Hinton agar using Agar diffusion techniques at concentrations of 100, 50 and 25 μg/mL, respectively. Muller Hinton agar (MHA) plates were inoculated with test inoculum (E. coli, Pseudomonas aeruginosa and Staphylococcus aureus) of standard inoculum (0.5 McFarland). Nalidixic acid and Imipenem were used as a reference drug for bacteria. Briefly, MHA agar plates were inoculated with bacterial strains under sterile conditions, and disc (diameter = 8 mm) was loaded with 50 μL of the tested sample. The plate was incubated at 37 °C for 24 hours. Nalidixic acid and Imipenem were used as a reference drug for bacteria. After the incubation period, the antimicrobial agent usually diffuses into the agar and inhibits the germination and growth of the test microorganism, and then according to the Clinical Laboratory Standards (CLSI), the diameter of the growth-inhibiting zone was measured^44^. The MICs of the prepared compounds were determined by the agar dilution plate technique following the standard procedure of the Clinical and Laboratory Standards^44^. The MICs were calculated from the X-axis intercept of the linear graph between log (Inhibitory concentration) and the growth inhibitory zone area.

The fungal strain was grown in 5 mL Sabourad Glucose Broth (glucose: peptone; 40: 10) for 3–4 days to reach 10^5^ CFU/mL cells. Fungal cultures (0.1 mL) were spread evenly on Sabourad dextrose agar plates. The plates were inoculated with fungal strains under sterile conditions, and disc (diameter = 8 mm) was loaded with 50 μL of the test sample. The plate was then incubated at 30 °C for 3–4 days and according to the Clinical Laboratory Standards (CLSI), the diameter of the growth-inhibiting zone was measured^44^. Nystatin was used as antifungal standard drug^45^.

##### Anti-inflammatory activity of the newly synthesized compounds

Carrageenan-induced paw edema method was used to evaluate the anti-inflammatory effect of the selected prepared compounds^46,47^. Indomethacin drug as a suspension in 24 tweens 80 was used as the reference drug, using Winter *et al*. method^46,47^. The inhibition percentage of inflammation was calculated using the following equation:

$$Inhibation\, \% =\frac{(weight\,of\,paw\,edema\,of\,control-weight\,of\,paw\,edema\,of\,related)\times 100}{weight\,of\,paw\,edema\,of\,control}$$

Microanalyses (CHNS elemental) and anti-inflammatory activity of the prepared compounds were carried out in the pharmaceutical Microbiology Department, National Center for Radiation Research and Technology (NCRRT), Nasr City, Egypt.

#### Molecular docking

Based on the results of antibacterial screening and the anti-inflammatory assay, docking of the most active compound **5d** and the positive control, **Nalidixic acid**, in case of antibacterial screening was performed with the binding site of DNA Gyrase (topoisomerase II) enzyme (PDB ID: 2XCT). Also, docking of compounds **10a,b, 12a,b** and the positive control **Indomethacin** in case of anti-inflammatory effect was performed with the binding site of COX-2 (PDB ID: 1CX2). Docking was done to shed light on their potential binding modes and investigate their similarity to the standard ligand binding modes^48–50^.

##### Molecular docking procedure

X-ray crystal structure of DNA Gyrase (topoisomerase II) complexed with a Ciprofloxacin was determined at 3.35 Å resolution and X-ray crystal structure of COX-2 complexed with a selective inhibitor, sc-558 at 3 Å resolution. All molecular modeling calculations and docking studies were carried out using ‘Molecular Operating Environment 2019.0101’ software (MOE of Chemical Computing Group Inc., on a Core i7 2.2 GHz workstation) running on a Windows 10 PC.

##### Preparation of the targets (DNA Gyrase (topoisomerase II) enzyme and COX-2)

The X-ray crystallographic structures of DNA Gyrase (topoisomerase II) enzyme (PDB ID: **2XCT**) and cyclo-oxygenase 2 (PDB ID: **1CX2**) were downloaded from the protein data bank (http://www.rcsb.org/)^51^. The enzyme was prepared for docking study by removal of chain B, C and D of its dimmers, water molecules, and ligands that are not involved in the binding. The enzyme was then prepared using quick preparation protocol in MOE with default options.

##### Docking validation

To confirm whether the applied docking protocol is valid or not, Re-docking of the co-crystallized ligand into the enzyme was done. Based on the binding mode and rmsd (root mean square deviation), the coordinates of the native ligand in the co-crystallized PDB file were compared with the coordinates of the greatest scoring docking pose of the native ligand. The docking validation results revealed a near-perfect alignment with the original ligand as attained from the resolved X-ray PDB file. In the case of antibacterial screening, docking validation was confirmed from the small root mean square deviation (rmsd = 0.3155) between the docked pose and the co-crystallized ligand (S (energy score) of −11.2658 kcal/mol). Docking validation was also demonstrated by the ability of the docking poses to restore the main interactions that occur between the co-crystallized ligand and the active site’s hot spots, Manganese (Mn) atom and DG 5 of DNA Gyrase enzyme. In case of anti-inflammatory effect, the re-docked ligand showed a small root mean square deviation value (rmsd = 0.0215) between the docked compound and the co-crystallized ligand (S of −15.569 kcal/mol), they also showed a high ability to repeat the main interactions that occur between the co-crystallized ligand and the active site’s hot spots, Arg513 and His90 of COX-II.

##### Active compounds preparation for docking

Active compounds preparation for docking was done as follows: firstly, Marvin Sketch was used to built up the 2D structures of the docked ligands and copied onto MOE. This step is followed by 3D protonation of the active compounds structure. Then the systemic search was used for the running of the conformational analysis and then the smallest energetic conformer was selected. An identical docking protocol was applied with the ligand.

##### Running of docking

Docking studies were performed using DNA Gyrase (topoisomerase II) enzyme co-crystallized with the native ligand of protein data bank file ID: **2XCT** (PDB ID: **2XCT**) and also COX-II enzyme co-crystallized with the native ligand of protein data bank file ID: **1CX2** (PDB ID: **1CX2**). Posing compounds **5d** and **Nalidixic acid**, in case of antibacterial screening, and compounds **10a,b, 12a,b** and the positive control **Indomethacin**, in case of anti-inflammatory effect, was scored by London dG scoring function, that was used for docking, and Triangle Matcher placement protocol. The docked compounds of the greatest scoring pose were documented. Interactions between ligand and receptor in the formed complexes were tested in both 3D and 2D styles.

## Results and Discussion

### The spectral data and physical constants of the newly synthesized compounds

- For compound (3a) (N^3^-(5-chloropyrimidin-2-yl)-5-phenyl-4H-1,2,4-triazole-3,4-diamine):
  Yield 68% crystal from dioxan; mp 213–215 ^o^C. Analysis calculated for C_12_H_10_ClN_7_ (287): C, 50.10; N, 34.08; H, 3.50; Cl, 12.32. The analyses found for the compound are: C, 50.16; H, 3.57; Cl, 12.30; N, 34.15). The UV spectrum gave [λ_max_ (Log ε)]: 372 nm. IR characteristic peaks appear at (ν cm^−1^): 3449, 3318 (NH_2_), 3189 (NH), 1601, 1581(C=N). ^1^H NMR characteristic peaks appear at δ ppm: 5.87(s, (2H), NH_2_), 4.0(s, (1H), NH), 8.05(s, 2H, (CH)-pyrimidine), 7.41–8.28 (m, 5H, CH-benzene). ^13^C NMR characteristic peaks appear at δ ppm: 119.0 (C5 of pyrimidine, C-Cl), 126.50,129.20 (C_2_ and C_3_ of benzene), 131.1 (C_4_ of benzene), 130.6 (C_1_ of benzene), 151.1 (C_5_ of triazole), 157.2 (C_2_ of triazole), 167.9 (C_2_ of pyrimidine), 156.8 (C_4 and_ C_6_ of pyrimidine). MS (Int.%): 287 (5.1, M^+^), 271(12.5, M^+^ - NH_2_), 174 (22.5, M^+^ - 4-chloropyrimidyl), 159 (34.8, M^+^ - 4-chloropyrimidyl-NH), 128 (15.1, 4-chloropyrimidyl-NH), 113 (12.2, 4-chloropyrimidyl).
- For compound (3b) (N^3^-(5-chloropyrimidin-2-yl)-5-(pyridin-4-yl)-4H-1,2,4-triazole-3,4-diamine):
  Yield 61% crystal from dioxan; mp 222–225 ^o^C. Analysis calculated for C_11_H_9_ClN_8_ (288): C, 45.76; H, 3.14; Cl, 12.28; N, 38.81. The analyses found for the compound are: C, 45.70; H, 3.11; Cl, 12.30; N, 38.83). The UV spectrum gave [λ_max_ (Log ε)]: 374 nm. IR characteristic peaks appear at (ν cm^−1^): 3442, 3325 (NH_2_), 3200 (NH), 1601,1581(C=N). ^1^H NMR characteristic peaks appear at δ ppm: 5.77(s, (2H), NH_2_), 4.0(s, (1H), NH), 8.05(s, 2H, (CH)-pyrimidine), 8.75(d, (2H), (CH)-pyridine), 7.99(d, (2H), (CH)-pyridine). ^13^C NMR characteristic peaks appear at δ ppm: 119.0 (C5 of pyrimidine, C-Cl), 121.3,149.8 (C_2_ and C_3_ of pyridine), 134 (C_4_ of pyridine), 157.2 (C_5_ of triazole), 151.1 (C_2_ of triazole), 167.9 (C_2_ of pyrimidine), 156.8 (C_4_ and C_6_ of pyrimidine). MS (Int.%): 288 (11.7, M^+^), 272(8.4, M^+^ - NH_2_), 175 (18.9, M^+^ - 4-chloropyrimidyl), 160 (31.2, M^+^ - 4-chloropyrimidyl-NH), 128 (13.4, 4-chloropyrimidyl-NH), 113 (11.8, 4-chloropyrimidyl).
- For compound (3c) (N^3^-(5-bromopyrimidin-2-yl)-5-phenyl-4H-1,2,4-triazole-3,4-diamine):
  Yield 52% crystal from dioxan; mp 288–291 ^o^C. Analysis calculated for C_12_H_10_BrN_7_ (331): C, 43.39; H, 3.03; Br, 24.06; N, 29.52. The analyses found for the compound are: C, 43.40; H, 3.01; Br, 24.08; N, 29.55). The UV spectrum gave [λ_max_ (Log ε)]: 370(0.80)nm. IR characteristic peaks appear at (ν cm^−1^): 3440, 3350 (NH_2_), 3195 (NH), 1600, 1578 (C=N). ^1^H NMR characteristic peaks appear at δ ppm: 5.71(s, (2H), NH_2_), 3.7(s, (1H), NH), 8.53(s, 2H, (CH)-pyrimidine), 7.04–8.25(m, 5H, CH-benzene). ^13^C NMR characteristic peaks appear at δ ppm: 110.7 (C5 of pyrimidine, C-Br), 127.5,129.2 (C_2_ and C_3_ of benzene), 131.1 (C_4_ of benzene), 130.6(C_1_ of benzene), 157.2 (C_5_ of triazole), 151.1 (C_2_ of triazole), 168.4 (C_2_ of pyrimidine), 159.1 (C_4 and_ C_6_ of pyrimidine). MS (Int.%): 331 (9.3, M^+^), 316 (9.1, M^+^ - NH_2_), 177 (22.3, M^+^ - 4-bromopyrimidyl), 162 (31.2, M^+^ - 4-bromopyrimidyl-NH), 170 (42.1, 4-bromopyrimidyl-NH), 155 (15.2, 4-bromopyrimidyl).
- For compound (3d) (N^3^-(5-bromopyrimidin-2-yl)-5-(pyridin-4-yl)-4H-1,2,4-triazole-3,4-diamine):
  Yield 48% crystal from dioxane; mp 257–259 ^o^C. Analysis calculated for C_11_H_9_BrN_8_ (332): C, 39.66; H, 2.72; Br, 23.98; N, 33.63 C, 39.66; H, 2.72; Br, 23.98; N, 33.63. The analyses found for the compound are: C, 39.62; H, 2.75; Br, 24.00; N, 33.65). The UV spectrum gave [λ_max_ (Log ε)]: 374(0.84) nm. IR characteristic peaks appear at (ν cm^−1^): 3452, 3367 (NH_2_), 3188 (NH), 1600,1568 (C=N). ^1^H NMR characteristic peaks appear at δ ppm: 5.68(s, (2H), NH_2_), 4.2(s, (1H), NH), 8.56(s, 2H, (CH)-pyrimidine), 8.82(d, (2H), (CH)-pyridine), 7.95(d, (2H), (CH)-pyridine). ^13^C NMR characteristic peaks appear at δ ppm: 110.7 (C5 of pyrimidine, C-Br), 121.3, 149.8 (C_2_ and C_3_ of pyridine), 134.0 (C_4_ of pyridine), 157.2 (C_5_ of triazole), 151.1 (C_2_ of triazole), 168.4 (C_2_ of pyrimidine), 159.1 (C_4_ and C_6_ of pyrimidine). MS (Int.%): 332 (13.8, M^+^), 317(11.1, M^+^ - NH_2_), 178 (19.8, M^+^ - 4-bromopyrimidyl), 163 (33.5, M^+^ - 4-bromopyrimidyl-NH), 170 (31.5, 4-bromopyrimidyl-NH).
- For compound (5a) (8-(5-chloropyrimidin-2-yl)-3,6,6-triphenyl-5,6,7,8-tetrahydro-[1,2,4]triazolo-[3,4-f] [1–3,5]triazaphosphinin-6-ol):
  Yield 56%, crystals from dioxan; mp 277–279 ^o^C. Analysis calculated for C_25_H_21_ClN_7_OP (501): C, 59.83; H, 4.22; Cl, 7.06; N, 19.53; P, 6.17. The analyses found for the compound are: C,59.80; H, 4.23; N,19.56; Cl, 7.08, P, 6.18. IR characteristic peaks appear at (ν cm^−1^): 3080(aromatic (CH)), 2925(aliphatic (CH)), 1650(P-OH), 1522(C=N). ^1^H NMR characteristic peaks appear at δ ppm: 2.8(s, 1H, OH), 2.0(s, (1H), NH), 3.1(s, (2H), CH_2_), 7.41–8.05(m, 15H, CH-benzene), 8.08(s, 2H, (CH)-pyrimidine). ^13^C NMR characteristic peaks appear at δ ppm: 119.0 (C_5_ of pyrimidine, C-Cl), 130.6 (C_1_ of benzene, C-triazole), 127.5 (C_2_ of benzene attached with triazole), 129.2 (C_3_ of benzene attached with triazole), 131.1 (C_4_ of benzene attached with triazole), 131.2 (C-P of benzene), 84 (N-C-P of triaziphosphine), 157.2 (C_5_ of triazole), 151.1 (C_2_ of triazole), 167.9 (C_2_ of pyrimidine), 156.6 (C_4_ and C_6_ of pyrimidine).
- For compound (5b) (8-(5-chloropyrimidin-2-yl)-3-(pyridine-4-yl)-6,6-diphenyl-5,6,7,8-tetrahydro[1,2,4]triazolo[3,4-f][1–3,5]triazaphosphinin-6-ol):
  Yield 43%, crystals from dioxan; mp 300–302 ^o^C. Analysis calculated for C_24_H_20_ClN_8_OP (502):C, 57.32; H, 4.01; Cl, 7.05; N, 22.28; O, 3.18; P, 6.16. The analyses found for the compound are: C, 57.40; H, 3.98; N,22.30; Cl, 7.07, P, 6.18. IR characteristic peaks appear at (ν cm^−1^): 3078(aromatic (CH)), 2943(aliphatic (CH)), 1647(P-OH), 1518(C=N). ^1^H NMR characteristic peaks appear at δ ppm: 3.1(s, 1H, OH), 2.3(s, (1H), NH), 3.6(s, (2H), CH_2_), 7.35–8.09(m, 10H, CH-benzene), 8.21(s, 2H, (CH)-pyrimidine), 8.75(d, (2H), (CH)-pyridine), 7.99(d, (2H), (CH)-pyridine). ^13^C NMR characteristic peaks appear at δ ppm: 119.0 (C5 of pyrimidine, C-Cl), 134(C_4_ of pyridine), 121.3 (C_3_ and C_5_ of pyridine), 149.8 (C_2_ and C_6_ of pyridine), 131.2 (C-P of benzene), 131.2 (C_2_ and C_6_ of benzene), 128.7 (C_3,4,5_ of benzene), 84.3 (N-C-P of triaziphosphine), 157.2 (C_2_ of triazole), 151.1 (C_5_ of triazole), 167.9 (C_2_ of pyrimidine), 156.6 (C_4_ and C_6_ of pyrimidine).
- For compound (5c) (8-(5-Bromopyrimidin-2-yl)-3,6,6-triphenyl-5,6,7,8-tetrahydro [1,2,4]triazolo[3,4-f] [1–3,5]triazaphosphinin-6-ol):
  Yield 51%, crystals from dioxan; mp 222–225 ^o^C. Analysis calculated for C_25_H_21_BrN_7_OP (554): C, 54.96; H, 3.87; Br, 14.62; N, 17.95; O, 2.93; P, 5.67. The analyses found for the compound are: C, 54.94; H, 3.86; Br, 14.65; N, 18.00; O, 2.93; P, 5.70. IR characteristic peaks appear at (ν cm^−1^): 3073(aromatic (CH)), 2952(aliphatic (CH)), 1643(P-OH), 1519 (C=N). ^1^H NMR characteristic peaks appear at δ ppm: 3.3(s, 1H, OH), 2.4(s, (1H), NH), 3.6(s, (2H), CH_2_), 7.30–8.05(m, 15H, CH-benzene), 8.34(s, 2H, (CH)-pyrimidine). ^13^C NMR characteristic peaks appear at δ ppm: 110.7 (C-Br of pyrimidine), 130.6 (C_1_ of benzene, C-triazole), 127.5 (C_2_ and C_6_ of benzene attached with triazole), 129.2 (C_3_ and C_5_ of benzene attached with triazole), 131.1 (C_4_ of benzene attached with triazole), 131.2 (C-P of benzene), 84.3 (N-C-P of triaziphosphine), 157.2 (C_5_ of triazole), 151.1 (C_2_ of triazole), 168.4 (C_2_ of pyrimidine, C-N), 159.1 (C_4_ and C_6_ of pyrimidine).
- For compound (5d) (8-(5-Bromopyrimidin-2-yl)-3-(4-pyridin-4-yl)-6,6-triphenyl-5,6,7,8-tetrahydro[1,2,4]triazolo[3,4-f][1–3,5]triazaphosphinin-6-ol):
  Yield 39%, crystals from dioxan; mp 267–269 ^o^C. Analysis calculated for C_24_H_20_BrN_8_OP (554): C, 54.96; H, 3.87; Br, 14.62; N, 17.95; O, 2.93; P, 5.67. The analyses found for the compound are: C, 54.94; H, 3.86; Br, 14.65; N, 18.00; O, 2.93; P, 5.70. IR characteristic peaks appear at (ν cm^−1^): 3073(aromatic (CH)), 2952(aliphatic (CH)), 1643(P-OH), 1519 (C=N). ^1^H NMR characteristic peaks appear at δ ppm: 3.3(s, 1H, OH), 2.4(s, (1H), NH), 3.6(s, (2H), CH_2_), 7.30–8.05(m, 15H, CH-benzene), 8.34(s, 2H, (CH)-pyrimidine). ^13^C NMR characteristic peaks appear at δ ppm: 110.7 (C-Br of pyrimidine), 130.6 (C_1_ of benzene, C-triazole), 127.5 (C_2_ and C_6_ of benzene attached with triazole), 129.2 (C_3_ and C_5_ of benzene attached with triazole), 131.1 (C_4_ of benzene attached with triazole), 131.2 (C-P of benzene), 84.3 (N-C-P of triaziphosphine), 157.2 (C_5_ of triazole), 151.1 (C_2_ of triazole), 168.4 (C_2_ of pyrimidine, C-N), 159.1 (C_4_ and C_6_ of pyrimidine).
- For compound (6a) (8-(5-chloropyrimidin-2-yl)-6-phenyl-3-(pyridin-2-yl)-7,8-dihydro-[1,2,4]triazolo-[4,3-b] [1,2,4]triazine):
  Yield 51%, crystals from dioxane/diluted by methanol, mp 171–173^o^C. Analysis calculated for C_19_H_13_ClN_8_ (388): C, 58.69; H, 3.37; Cl, 9.12; N, 28.82. The analyses found for the compound are: C, 58.7; H, 3.35; N, 28.9; Cl, 9.15. IR characteristic peaks appear at (ν cm^−1^): 3079.20, 2966 cm^−1^ (aromatic & aliphatic (CH)), 1522(C=N). ^1^H NMR characteristic peaks appear at δ ppm: 3.13(s, (2H), CH_2_) triazine ring, 8.05(s, 2H, (CH)-pyrimidine), 7.52–7.94(m, 5H, CH-benzene), 8.38 (d, 1H, CH- pyridine), 8.59(d, 1H, CH-pyridine), 7.36(dd, 1H, CH-pyridine), 7.85(dd, 1H, CH-pyridine). ^13^C NMR characteristic peaks appear at δ ppm: 134.7 (C_1_ of benzene), 128.2 (C_2,6_ of benzene), 128.8 (C_3_ and C_5_ of benzene), 131.0 (C_4_ of benzene), 157.2 (C_5_ of triazole), 151.1 (C_2_ of triazole), 164.6 (C_6_ of Triazine), 62.0 (C_5_ of Triazine),156.8 (C_4_ and C_6_ of pyrimidine), 119 (pyrimidine C-Cl), 155 (C_2_ pyridine, C-N), 124.2 (C_3_ pyridine), 137.2 (C_4_ pyridine), 123.6 (C_5_ pyridine), 149.2 (C_6_ pyridine).
- For compound (6b) (8-(5-chloropyrimidin-2-yl)-6-phenyl-3-(pyrimidin-4-yl)-7,8-dihydro[1,2,4]triazolo-[4,3-b] [1,2,4]triazine):
  Yield 41%, crystals from dioxan/diluted by methanol, mp 188–191 ^o^C. Analysis calculated for C_18_H_12_ClN_9_ (389): C, 55.46; H, 3.10; Cl, 9.10; N, 32.34. The analyses found for the compound are: C, 55.5; H, 3.08; N, 32.3; Cl, 9.2. IR characteristic peaks appear at (ν cm^−1^): 3078, 2959 cm^−1^ (aromatic & aliphatic (CH)), 1519 (C=N). ^1^H NMR characteristic peaks appear at δ ppm: 3.4(s, (2H), CH_2_, triazine ring), 8.08 (s, 2H, (CH)-pyrimidine), 7.52–7.94(m, 5H, CH-benzene), 8.29 (d, 1H, CH- pyrimidine), 9.36(s, 1H, CH- pyrimidine), 9.20(d, 1H, CH- pyrimidine). ^13^C NMR characteristic peaks appear at δ ppm: 134.0 (C_1_ of benzene), 128.2 (C_2,6_ of benzene), 128.8 (C_3_ and C_5_ of benzene), 131.0 (C_4_ of benzene), 157.2 (C_5_ of triazole), 151.1 (C_2_ of triazole), 164.6 (C_6_ of triazine), 62.0 (C_5_ of triazine),156.8 (C_4_ and C_6_ of pyrimidine), 119 (pyrimidine C-Cl), 167.9 (C_2_ pyrimidine, C-N), 164 (C_4_ non-substituted pyrimidine), 115.6 (C_5_ of non-substituted pyrimidine), 157.1 (C_6_ of non-substituted pyrimidine).
- For compound (6c) (8-(5-bromopyrimidin-2-yl)-6-phenyl-3-(pyridin-2-yl)-7,8-dihydro-[1,2,4]triazolo-[4,3-b] [1,2,4]triazine):
  Yield 36%, crystals from dioxan/diluted by methanol, mp 157–160 ^o^C. Analysis calculated for C_19_H_13_BrN_8_ (432): C, 52.67; H, 3.02; Br, 18.44; N, 25.86. The analyses found for the compound are: C, 52.7; H, 3.01; N, 25.9; Br, 18.5. IR characteristic peaks appear at (ν cm^−1^): 3089, 2977 cm^−1^ (aromatic & aliphatic (CH)), 1520 (C=N). ^1^H NMR characteristic peaks appear at δ ppm: 3.3 (s, (2H), CH_2_, triazine ring), 8.5 (s, 2H, (CH)-pyrimidine), 7.4–7.8 (m, 5H, CH-benzene), 8.8 (d, 1H, CH- pyridine), 8.9 (d, 1H, CH-pyridine), 7.6 (dd, 1H, CH-pyridine), 7.8 (dd, 1H, CH-pyridine). ^13^C NMR characteristic peaks appear at δ ppm: 134.0 (C_1_ of benzene), 128.2 (C_2,6_ of benzene), 128.8 (C_3_ and C_5_ of benzene), 131.0 (C_4_ of benzene), 157.2 (C_5_ of triazole), 151.1 (C_2_ of triazole), 164.6 (C_6_ of triazine), 62.0 (C_5_ of triazine),159.1 (C_4_ and C_6_ of pyrimidine), 110.7 (pyrimidine C-Br), 168.4 (C_2_ pyrimidine, C-N), 155 (C_2_ pyridine, C-N), 124.2 (C_3_ pyridine), 137.2 (C_4_ of pyridine), 123.5 (C_5_ of pyridine), 149.2 (C_6_ of pyridine).
- For compound (6d) (8-(5-bromopyrimidin-2-yl)-6-phenyl-3-(pyrimidin-4-yl)-7,8-dihydro-[1,2,4]triazolo-[4,3-b][1,2,4]triazine):
  Yield 49%, crystals from dioxan/diluted by methanol, mp 189–191 ^o^C. Analysis calculated for C_18_H_12_BrN_9_ (433): C, 49.79; H, 2.79; Br, 18.40; N, 29.03. The analyses found for the compound are: C, 49.7; H, 2.8; N, 29.1; Br, 18.4. IR characteristic peaks appear at (ν cm^−1^): 3078, 2959 cm^−1^ (aromatic & aliphatic (CH)), 1522 (C=N). ^1^H NMR characteristic peaks appear at δ ppm: 3.5 (s, (2H), CH_2_, triazine ring), 8.4 (s, 2H, (CH)-pyrimidine), 7.5–7.9 (m, 5H, CH-benzene), 8.17 (d, 1H, CH- pyrimidine), 9.36(s, 1H, CH- pyrimidine), 9.20(d, 1H, CH- pyrimidine). ^13^C NMR characteristic peaks appear at δ ppm: 134.0 (C_1_ of benzene), 128.2 (C_2,6_ of benzene), 128.8 (C_3_ and C_5_ of benzene), 131.0 (C_4_ of benzene), 157.2 (C_5_ of triazole), 151.1 (C_2_ of triazole), 164.6 (C_6_ of triazine), 62.0 (C_5_ of triazine),159.1 (C_4_ and C_6_ of pyrimidine), 110.7 (pyrimidine C-Br), 168.4 (C_2_ pyrimidine, C-N), 167.9 (C_2_ pyrimidine, C-N), 164 (C_4_ pyrimidine, C-N), 115.5 (C_5_ of non-substituted pyrimidine), 157.1 (C_6_ of non-substituted pyrimidine).
  For compound (7a) (8-(5-chloropyrimidin-2-yl)-6-methyl-3-phenyl-[1,2,4]triazolo[4,3-b][1,2,4]triazin-7(8H)-one):
  Yield 55% crystal from ethanol; mp 300–303 ^o^C. Analysis calculated for C_15_H_10_ClN_7_O (339): C, 53.03; H, 2.97; Cl, 10.44; N, 28.86. The analyses found for the compound are: C,53.02; H, 3.0; N, 28.9; Cl, 10.4. IR characteristic peaks appear at (ν cm^−1^): 3089, 2988 cm^−1^ (aromatic & aliphatic (CH)), 1725 (C=O), 1590 (C=N). ^1^H NMR characteristic peaks appear at δ ppm: 2.07(s, 3H, CH_3_), 8.05(s, 2H, (CH)-pyrimidine), 7.41–8.28(m, 5H, CH-benzene). MS (m/z, %): 339 (M+, 100.0%), 341 (M + 2, 32.4%), 225 (M + - 5-chloropyrimidine, 13.4%), 227 ((M + 2)-5-chloropyrimidine, 4.8%), 114 (5-chloropyrimidine, 32%), 116 (5-chloropyrimidine, 10.3%).
- For compound (7b) (8-(5-chloropyrimidin-2-yl)-6-methyl-3-(pyridin-4-yl)-[1,2,4]triazolo[4,3-b][1,2,4]-triazin-7(8H)-one):
  Yield 39% crystal from ethanol; mp 289–292 ^o^C. Analysis calculated for C_14_H_9_ClN_8_O (340): C, 49.35; H, 2.66; Cl, 10.41; N, 32.89. The analyses found for the compound are: C,49.4; H, 2.7; N, 32.9; Cl, 10.4. IR characteristic peaks appear at (ν cm^−1^): 3091, 2979 cm^−1^ (aromatic & aliphatic (CH)), 1728 (C=O), 1522 (C=N). ^1^H NMR characteristic peaks appear at δ ppm: 2.18 (s, 3H, CH_3_), 8.02 (s, 2H, (CH)-pyrimidine), 7.99(d, (2H), (CH)-pyridine), 8.75(d, (2H), (CH)-pyridine). MS (m/z, %): 340 (M+, 100.0%), 342 (M^+^ + 2, 32.4%), 226 (M + - 5-chloropyrimidine, 12.3%), 228 ((M + 2) - 5-chloropyrimidine, 4.1%), 114 (5-chloropyrimidine, 15.3%), 116 (5-chloropyrimidine, 5.1%).
- For compound (7c) (8-(5-bromopyrimidin-2-yl)-6-methyl-3-(pyridin-4-yl)-[1,2,4]triazolo[4,3-b][1,2,4]-triazin-7(8H)-one):
  Yield 42% crystal from ethanol; mp 244–246 ^o^C. Analysis calculated for C_15_H_10_BrN_7_O (383): C, 46.89; H, 2.62; Br, 20.80; N, 25.52. The analyses found for the compound are: C,47.0; H, 2.6; N, 25.5; Br, 20.8. IR characteristic peaks appear at (ν cm^−1^): 3077, 2987 cm^−1^ (aromatic & aliphatic (CH)), 1724 (C=O), 1520 (C=N). ^1^H NMR characteristic peaks appear at δ ppm: 2.23 (s, 3H, CH_3_), 8.78 (s, 2H, (CH)-pyrimidine), 7.41–8.22 (m, 5H, CH-benzene). MS (m/z, %): 383 (M^+^, 100.0%), 385 (M + 2, 33.4%), 225 (M^+^ - 5-bromopyrimidine, 9.6%), 227 ((M + 2) - 5-bromopyrimidine, 3.2%), 158 (5-bromopyrimidine, 21.3%), 160 (5-bromopyrimidine, 7.1%).
- For compound (7d) (8-(5-chloropyrimidin-2-yl)-3-phenyl-[1,2,4]triazolo[4,3-b][1,2,4]triazine-6,7(5H,8H)-dione):
  Yield 36% crystal from ethanol; mp 274–276 ^o^C. Analysis calculated for C_14_H_9_BrN_8_O (384): C, 43.66; H, 2.36; Br, 20.74; N, 29.09. The analyses found for the compound are: C,43.7; H, 2.4; N, 29.1; Br, 20.7. IR characteristic peaks appear at (ν cm^−1^): 3079, 2977 cm^−1^ (aromatic & aliphatic (CH)), 1730 (C=O), 1518 (C=N). ^1^H NMR characteristic peaks appear at δ ppm: 2.17(s, 3H, CH_3_), 8.43(s, 2H, (CH)-pyrimidine), 7.95 (d, (2H), (CH)-pyridine), 8.78 (d, (2H), (CH)-pyridine). MS (m/z, %): 384 (M+, 100.0%), 386 (M + + 2, 33.2%), 226 (M + - 5-bromopyrimidine, 11.7%), 228 ((M + 2) - 5-bromopyrimidine, 3.8%), 159 (5-bromopyrimidine, 17.5%), 161 (5-bromopyrimidine, 5.8%).
- For compound (8a) (8-(5-chloropyrimidin-2-yl)-3-(pyridin-4-yl)-[1,2,4]triazolo[4,3-b][1,2,4]triazine-6,7(5H,8H)-dione):
  Yield 42% crystalized from ethanol; mp 152–155 ^o^C. Analysis calculated for C_14_H_8_ClN_7_O_2_ (341): C, 49.21; H, 2.36; Cl, 10.38; N, 28.69. The analyses found for the compound are: C, 49.2; H, 2.4; N, 28.7, Cl, 10.4. IR characteristic peaks appear at (ν cm^−1^): 3233 (NH), 3079 (aromatic (CH)), 1734, 1658 (2C=O), 1522 (C=N). ^1^H NMR characteristic peaks appear at δ ppm: 7.6 (s, (1H), NH), 8.05 (s, 2H, (CH)-pyrimidine), 7.03–8.28 (m, 5H, CH-benzene).
- For compound (8b) (8-(5-bromopyrimidin-2-yl)-3-phenyl-[1,2,4]triazolo[4,3-b][1,2,4]triazine-6,7(5H,8H)-dione):
  Yield 38% crystalized from ethanol; mp 215–217 ^o^C. Analysis calculated for C_13_H_7_ClN_8_O_2_ (342): C, 45.56; H, 2.06; Cl, 10.35; N, 32.70. The analyses found for the compound are: C, 45.6; H, 2.1; N, 32.7, Cl, 10.3. IR characteristic peaks appear at (ν cm^−1^): 3197 (NH), 1729, 1666 (C=O), 1520 (C=N). ^1^H NMR characteristic peaks appear at δ ppm: 7.9 (s, (1H), NH), 8.12 (s, 2H, (CH)-pyrimidine), 7.96 (d, (2H), (CH)-pyridine), 8.05 (d, (2H), (CH)-pyridine).
- For compound (8c) (8-(5-bromopyrimidin-2-yl)-3-phenyl-[1,2,4]triazolo[4,3-b][1,2,4]triazine-6,7(5H,8H)-dione):
  Yield 52% crystalized from ethanol/acetic acid mixture; mp 197–199 ^o^C. Analysis calculated for C_14_H_8_BrN_7_O_2_ (384): C, 43.54; H, 2.09; Br, 20.69; N, 25.39. The analyses found for the compound are: C, 43.5; H, 2.1; N, 25.4, Br, 20.7. IR characteristic peaks appear at (ν cm^−1^): 3188 (NH), 3080 (aromatic (CH)), 1731, 1656 (C=O), 1519 (C=N). ^1^H NMR characteristic peaks appear at δ ppm: 7.81 (s, (1H), NH), 8.42 (s, 2H, (CH)-pyrimidine), 7.45–8.06 (m, 5H, CH-benzene).
- For compound (8d) (8-(5-bromopyrimidin-2-yl)-3-(pyridin-4-yl)-[1,2,4]triazolo[4,3-b][1,2,4]triazine-6,7(5H,8H)-dione):
  Yield 57% crystalized from diluted ethanol; mp 223–225 ^o^C. Analysis calculated for C_13_H_7_BrN_8_O_2_ (385): C, 40.33; H, 1.82; Br, 20.64; N, 28.94. The analyses found for the compound are: C, 40.3; H, 1.8; N, 28.9, Br, 20.6. IR characteristic peaks appear at (ν cm^−1^): 3180 (NH), 3088 (aromatic (CH)), 1735, 1668 (C=O), 1520 (C=N). ^1^H NMR characteristic peaks appear at δ ppm: 7.82 (s, (1H), NH), 8.43 (s, 2H, (CH)-pyrimidine), 7.87 (d, (2H), (CH)-pyridine), 8.15 (d, (2H), (CH)-pyridine).
- For compound (9a) (3-hydrazinyl-5-phenyl-4H-1,2,4-triazol-4-amine):
  Yield 67% crystal from ethanol; mp 155–157 ^o^C. Analysis calculated for C_8_H_10_N_6_ (190): C, 50.52; H, 5.30; N, 44.18. The analyses found for the compound are: C, 50.5; H, 5.3; N, 44.2. UV [DMF, λ _max_ nm (Log ε)]: 316 (2.4) nm. IR characteristic peaks appear at (ν cm^−1^): 3344, (NH_2_), 3174 (NH), 3076 (aromatic (CH)), 1602 (deformation NH_2_), 1522 (C=N). ^1^H NMR characteristic peaks appear at δ ppm: 4.11 (s, (2H), NH_2_ of hydrazine), 5.77 (s, (2H), NH_2_ of triazole), 7.41–8.08 (m, 5H, CH-benzene), 9.23 (s, 1H, NH of hydrazine).
- For compound (9b)(3-hydrazinyl-5-(pyridin-4-yl)-4H-1,2,4-triazol-4-amine):
  Yield 58% crystal from ethanol; mp 187–189 ^o^C. Analysis calculated for C_7_H_9_N_7_ (191): C, 43.97; H, 4.74; N, 51.28. The analyses found for the compound are: C, 44.0; H, 4.7; N, 51.3. UV [DMF, λ _max_ nm (Log ε)]: 312 (2.1) nm. IR characteristic peaks appear at (ν cm^−1^): 3356, (NH_2_), 3181 (NH), 1600 (deformation NH_2_), 1518 (C=N). ^1^H NMR characteristic peaks appear at δ ppm: 4.23 (s, (2H), NH_2_ of hydrazine), 5.53 (s, (2H), NH_2_ of triazole), 7.83 (d, (2H), (CH)-pyridine), 8.87 (d, (2H), (CH)-pyridine), 9.76 (s, (1H), NH).
- For compound (10a) (7-phenyl-3,4-dihydro-[1,2,4]triazolo[4,3-e][1–5] tetrazaphosphinine):
  Yield 58% crystal from THF; mp 267–269 ^o^C. Analysis calculated for C_8_H_7_N_6_P (218): C, 44.04; H, 3.23; N, 38.52; P, 14.20. The analyses found for the compound are: C, 44.0; H, 3.3; N, 38.50; P, 14.2. IR characteristic peaks appear at (ν cm^−1^): 3123–2962 (broad, NH-NH), 1650 (P-NH), 1528 (C=N); ^1^H NMR characteristic peaks appear at δ ppm: 4.25 (s, 1H, NH-N), 6.32 (s, 1H, NH-P), 7.41–8.28 (m, 5H, CH-benzene). MS (m/z, %): 218 (M + ,4.34%), 194 (M^+^ - NH-NH, 100%), 163 (M^+^ - NH-NH-P, 44%), 148 (M^+^ - NH-NH-P-N, 2.21%).
- For compound (10b) (7-(pyridin-4-yl)-3,4-dihydro-[1,2,4]triazolo[4,3-e][1–5] tetrazaphosphinine):
  Yield 44% crystal from THF; mp 226–228 ^o^C. Analysis calculated for C_7_H_6_N_7_P (219): C, 38.37; H, 2.76; N, 44.74; P, 14.13. The analyses found for the compound are: C, 38.4; H, 2.8; N, 44.8; P, 14.1. IR characteristic peaks appear at (ν cm^−1^): 3120–2965 (broad, NH-NH), 1643 (P-NH), 1522 (C=N). ^1^H NMR characteristic peaks appear at δ ppm: 4.31 (s, 1H, NH-N), 6.52 (s, 1H, NH-P), 7.94 (d, (2H), (CH)-pyridine), 8.79 (d, (2H), (CH)-pyridine). MS (m/z, %): 219 (M^+^,6.22%), 195 (M^+^ - NH-NH, 100%), 164 (M^+^ - NH-NH-P, 37%), 149 (M^+^ - NH-NH-P-N, 5.11%).
- For compound (11a) (3-phenyl-1,7-dihydro-[1,2,4]triazolo[4,3-b][1,2,4,5]tetrazine):
  Yield 37% crystallized from dioxane; mp 252–254 ^o^C. Analysis calculated for C_9_H_8_N_6_ (200): C, 53.99; H, 4.03; N, 41.98. The analyses found for the compound are: C, 54.0; H, 4.0; N, 42.0. IR characteristic peaks appear at (ν cm^−1^): 3184,3132 (NH, NH of tetrazine and triazole), 1521 (C=N). ^1^H NMR characteristic peaks appear at δ ppm: 3.41 (s, 1H, NH-tetrazine), 5.82 (s, br., 1H, NH-triazole), 7.02 (s, 1H, CH-tetrazine), 7.51–7.82 (m, 5H, CH-benzene).
- For compound (11b) (3-(pyridin-4-yl)-1,7-dihydro-[1,2,4]triazolo[4,3-b][1,2,4,5]tetrazine):
  Yield 41% crystallized from dioxan; mp 271–273 ^o^C. Analysis calculated for C_8_H_7_N_7_ (201): C, 47.76; H, 3.51; N, 48.73. The analyses found for the compound are: C, 47.7; H, 3.5; N, 48.7. IR characteristic peaks appear at (ν cm^−1^): 3188, 3129 (NH, NH of tetrazine and triazole), 1519 (C=N). ^1^H NMR characteristic peaks appear at δ ppm: 3.62 (s, br., 1H, NH-tetrazine), 6.21 (s, br., 1H, NH-triazole), 7.36 (s, 1H, CH-tetrazine), 7.90 (d, (2H), (CH)-pyridine), 8.63 (d, (2H), (CH)-pyridine).
- For compound (12a) (6-(2-chloro-6-fluorophenyl)-3-phenyl-5,6,7,8-tetrahydro-[1,2,4]triazolo[4,3-b][1,2,4,5]tetrazine):
  Yield 53% crystal from dioxan; mp 234–236 ^o^C. Analysis calculated for C_15_H_12_ClFN_6_ (330): C, 54.47; H, 3.66; Cl, 10.72; F, 5.74; N, 25.41. The analyses found for the compound are: C, 54.5; H, 3.7; N, 25.4; F, 5.7; Cl, 10.7. UV [DMF, λ _max_ nm (Log ε)]: 265 (1.13) and 263 (1.03) nm. IR characteristic peaks appear at (ν cm^−1^): 3354,3169 and 3132 cm^−1^ (3NH), 3078 (aromatic (CH)), 1522 cm^−1^ (C=N). ^1^H NMR characteristic peaks appear at δ ppm: 2.31 (s, 1H, NH-tetrazine at 2-position), 4.32 (s, 1H, NH-tetrazine at 4-position), 5.04 (s, 1H, CH-tetrazine), 6.32 (s, 1H, NH-tetrazine at 5-position), 7.07–8.28 (m, 8H, CH-aromatic). ^13^C NMR characteristic peaks appear at δ ppm: 157.2 (C_3_ of triazole), 151.1 (C_5_ of triazole), 130.6 (C_1_ of non-substituted benzene), 127.5 (C_2_, C_6_ of non-substituted benzene), 129.2 (C_3_, C_5_ of non-substituted benzene), 131.1 (C_4_ of non-substituted benzene), 73.5 (C_3_ of tetrazine), 133.8 (C-Cl), 160.8 (C-F), 129.8 (C_2_ of substituted benzene), 124.2, 129.7, 113.4 (3 C of substituted benzene).
- For compound (12b) (6-(2-chloro-6-fluorophenyl)-3-(pyridin-4-yl)-5,6,7,8-tetrahydro-[1,2,4]triazolo[4,3-b][1,2,4,5] tetrazine):
  Yield 46% crystal from dioxan; mp 256–258 ^o^C. Analysis calculated for C_14_H_11_ClFN_7_ (331): C, 50.69; H, 3.34; Cl, 10.69; F, 5.73; N, 29.56. The analyses found for the compound are: C, 50.7; H, 3.4; N, 29.5; F, 5.7; Cl, 10.6. UV [DMF, λ _max_ nm (Log ε)]: 263(1.11) and 261 (1.01) nm. IR characteristic peaks appear at (ν cm^−1^): 3343, 3154 and 3129 cm^−1^ (3NH), 3072 (aromatic (CH)), 1518 cm^−1^ (C=N). ^1^H NMR characteristic peaks appear at δ ppm: 2.42 (s, 1H, NH-tetrazine at 2-position), 4.51 (s, 1H, NH-tetrazine at 4-position), 5.32 (s, 1H, CH-tetrazine), 6.50 (s, 1H, NH-tetrazine at 5-position), 7.01–7.32 (m, 3H, CH-aromatic), 7.92 (d, (2H), (CH)-pyridine), 8.71 (d, (2H), (CH)-pyridine). ^13^C NMR characteristic peaks appear at δ ppm: 157.2 (C_3_ of triazole), 151.1 (C_5_ of triazole), 134.0 (C4 of pyridine), 121.3 (C_3_, C_5_ of pyridine), 149.8 (C_2_, C_6_ of pyridine), 73.5 (C_3_ of tetrazine), 133.8 (C-Cl), 160.8 (C-F), 129.8 (C_2_ of substituted benzene), 124.2, 129.7, 113.4 (3C of substituted benzene).
- For compound (13a) (3-phenyl-7,8-dihydro-[1,2,4]triazolo[4,3-b][1,2,4,5]tetrazine-6(5H)-thione):
  Yield 45% crystal from THF; mp 276–278 ^o^C. Analysis calculated for C_9_H_8_N_6_S (232): C, 46.54; H, 3.47; N, 36.18; S, 13.81. The analyses found for the compound are: C, 46.5; H, 3.5; N, 36.2; S, 13.8. UV [λ_max_ (Log ε)]: 314 (2.1) nm. IR characteristic peaks appear at (ν cm^−1^): 3332, 3178 (NH-NH), 1528 (C=S). ^1^H NMR characteristic peaks appear at δ ppm: 3.8 (s, br., 1H, -NH- tetrazine at position 2), 5.3 (s, br., 1H, NH-tetrazine at position 4), 6.1 (s, br., 1H, NH-tetrazine at position 5), 7.52–8.07 (m, 5H of benzene). ^13^C NMR characteristic peaks appear at δ ppm: 157.2 (C_3_ of triazole), 151.1 (C_5_ of triazole), 182.1 (C_3_ of tetrazine), 130.6 (C_1_ of benzene), 127.5 (C_2,6_ benzene), 129 (C_3,5_ of benzene), 131.1 (C_4_ of benzene).
- For compound (13b) (3-(pyridin-4-yl)-7,8-dihydro-[1,2,4]triazolo[4,3-b][1,2,4,5]tetrazine-6(5H)-thione):
  Yield 51% crystal from THF; mp 255–258 ^o^C. Analysis calculated for C_8_H_7_N_7_S (233): C, 41.19; H, 3.02; N, 42.03; S, 13.75. The analyses found for the compound are: C, 41.2; H, 3.0; N, 42.0; S, 13.8. UV [λ_max_ (Log ε)]: 312 (1.8) nm. IR characteristic peaks appear at (ν cm^−1^): 3325, 3182 (NH-NH), 1522 (C=S). ^1^H NMR characteristic peaks appear at δ ppm: 3.2 (s, br., 1H, -NH- tetrazine at position 2), 5.1 (s, br., 1H, NH-tetrazine at position 4), 5.9 (s, br., 1H, NH-tetrazine at position 5), 7.99 (d, 2H of pridine at C_3_, C_5_), 8.77 (d, 2H of pyridine at C_2_, C_6_). ^13^C NMR characteristic peaks appear at δ ppm: 157.2 (C_3_ of triazole), 151.1 (C_5_ of triazole), 182.1 (C_3_ of tetrazine), 134.0 (C_4_ of pyridine), 149.8 (C_2,6_ of pyridine), 121.3 (C_3,5_ of pyridine).
- For compound (14a) (3-phenyl-7,8-dihydro-[1,2,4]triazolo[4,3-b][1,2,4,5]tetrazin-6(5H)-one):
  Yield 43% crystal from ethanol; mp 191–193 ^o^C. Analysis calculated for C_9_H_8_N_6_O (216): C, 50.00; H, 3.73; N, 38.87. The analyses found for the compound are: C, 50.1; H, 3.7; N, 38; 9. UV [λ _max_ (Log ε)]: 324 (1.51) nm. IR characteristic peaks appear at (ν cm^−1^): 3167 (NH-NH), 1643 (amidic CO), 1563 (C=N); ^1^H NMR characteristic peaks appear at δ ppm: 2.9 (s, br., 1H, NH- tetrazine at position 2), 4.8 (s, br., 1H, NH-tetrazine at position 4), 5.6 (s, br., 1H, NH-tetrazine at position 5), 7.87–8.08 (m, 5H of benzene). ^13^C NMR characteristic peaks appear at δ ppm: 157.2 (C_3_ of triazole), 151.1 (C_5_ of triazole), 152.4 (C_3_ of tetrazine), 130.6 (C_1_ of benzene), 127.5 (C_2,6_ of benzene), 129.2 (C_3,5_ of benzene), 131.1 (C4 of benzene). MS (m/z, %): 216 (M^+^, 4.7%), 188 (M^+^ - CO, 100%), 173 (M^+^ - CO-NH, 4.3%).
- For compound (14b) (3-(pyridin-4-yl)-7,8-dihydro-[1,2,4]triazolo[4,3-b][1,2,4,5]tetrazin-6(5H)-one):

Yield 38% crystal from ethanol; mp 170–173 ^o^C. Analysis calculated for C_8_H_7_N_7_O (217): C, 44.24; H, 3.25; N, 45.14. The analyses found for the compound are: C, 44.2; H, 3.3; N, 45.1. UV [λ _max_ (Log ε)]: 320 (1.21) nm. IR characteristic peaks appear at (ν cm^−1^): 3172 (NH-NH), 1651 (amidic CO), 1534 (C=N). ^1^H NMR characteristic peaks appear at δ ppm: 3.1 (s, br., 1H, -NH- tetrazine at position 2), 5.1 (s, br., 1H, NH-tetrazine at position 4), 5.9 (s, br., 1H, NH-tetrazine at position 5), 7.98 (d, 2H of pyridine at C_2,6_), 8.67 (d, 2H, of pyridine at C_3,5_). ^13^C NMR characteristic peaks appear at δ ppm: 157.2 (C_3_ of triazole), 151.1 (C_5_ of triazole), 152.4 (C_3_ of tetrazine), 134.0 (C_4_ of pyridine), 149.8 (C_2,6_ of pyridine), 121.3 (C_3,5_ of pyridine). MS (m/z, %): 217 (M^+^, 6.2%), 189 (M^+^ - CO, 100%), 174 (M^+^ - CO-NH, 3.7%).

### Samples characterization

**Figure 1:**
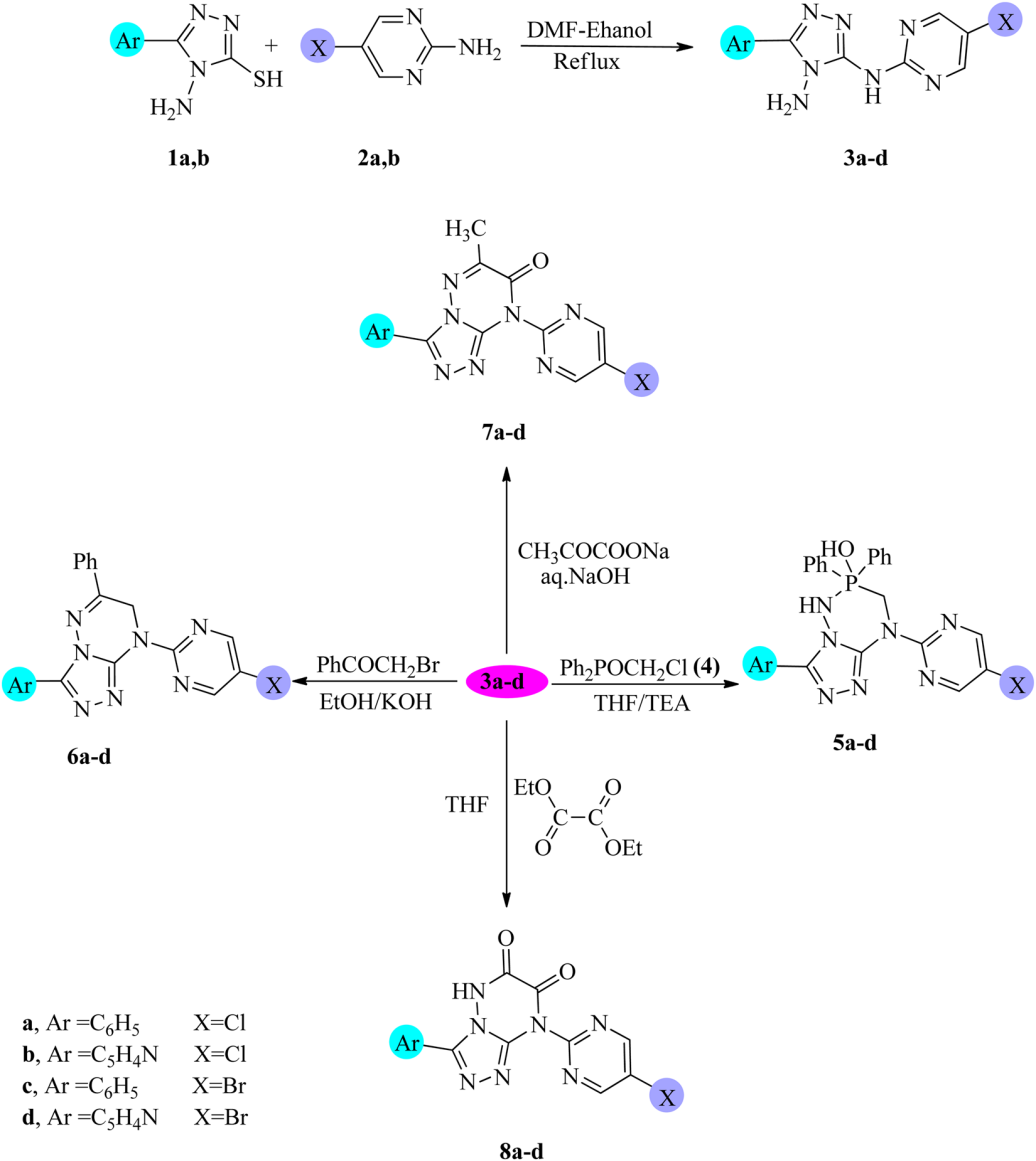
Synthesis of triazolotriazine derivatives **5–8**.

From the above mentioned spectral data and physical constants of the newly synthesized compounds, we concluded that: the mercapto group in the 4-amino-5-phenyl-4H-1,2,4-triazole-3-thiol (**1a**) is simply displaced by the amino group (nucleophilic group) in heterocyclic primary amines such as 5-chloropyrimidin-2-amine (**2a**) in Ethanol-DMF mixture (1:1) under reflux to yield 4-amino-5-substituted amino-1,2,4-triazole derivative (**3a**). Structure of **3a** was elucidated mainly from the disappearance of the mercapto group peak at 2600–2500 cm^−1^ in the IR spectrum, the new NH proton with NH_2_ proton of aminotriazole of compound **3a** showed through the ^1^H NMR spectrum at δ 14.22 ppm 5.87 ppm respectively. Also, the UV absorption of **3a** shows λ _max_ at 372 nm while the λ _max_ of **1a** appears at 317 nm, which proves the structure of compound **3a** (Fig. 1). Ring closure reactions^39^ of compound **3a** with diphenyl(chloromethyl)-phosphanoxide **(4)** under reflux in tetrahydrofuran (THF) produces 3,8-diaryl-4,5,6,7-tetrahydro-6-hydroxy-1,2,4-triazole[4,3-*b*][1–3,5]-phosphotriazine (**5a**) (Fig. 1). The presence of C-H, P-OH, P-N and NH, P-N, P-OH, and C-H functional groups peaks at 1522, 1650, 2925 and 3080 cm^−1^ in its IR spectrum were used to deduce the structure of **5a**. This structure was confirmed also by ^1^H NMR spectrum which showed resonated signals at δ 2.8 (s, 1H, OH), 3.1 (s, (2H), CH_2_), 2.0 (s, 1H, N-H), 7.41–8.05 (m, 15H, CH-benzene) and 8.08 (s, 2H, (CH)-pyrimidine).

Derivatives of 1,2,4-Triazolo[4,3-*b*]-1,2,4-triazine compounds (**6a–8a**) have been obtained from the hetero-cyclization of compound **3a** with phenacyl bromide dissolved in ethanolic potassium hydroxide, pyruvic acid dissolved sodium hydroxide and diethyloxalate dissolved in DMF/THF under reflux, respectively (Fig. 1). The chemical structures of compounds **6a–8a** were elucidated from both the spectral measurements and the elemental analyses. Infrared spectrum analysis of compound **6a** showed representative bands at ν 3079 (cm^−1^) and 2966 (cm^−1^) for aromatic & aliphatic (CH) and band at 1522 for (C=N). While, representing bands of compound **7a** were recorded at ν = 2978 (cm^−1^), 1480 (cm^−1^) for (stretching and bending vibration of CH_3_) and a band at 1725(cm^−1^) for (C=O). In addition to that compound **8a** bands were recorded at ν 3233 (cm^−1^) for NH, 1734(cm^−1^) and 1658(cm^−1^) for (2C=O), C=N and substituted pyrimidine and aromatic rings bands appear at 1522(cm^−1^) and 850–730 (cm^−1^). Also, 4 amino-5-(4 pyridyl)-4 *H*-1,2,4-triazole-3-thiol compound (**1b**) reacted with 5-chloro-pyrimidine-2-amine **(2b)** in Ethanol/DMF mixture (1:1) under reflux to give 4-amino-5-substituted amino-1,2,4-triazole derivative **3b. Compound 3b** in turn, undergo ring closure with each of diphenyl (chloromethyl) - phosphanoxide **(4)** in THF/TEA, phenacyl bromide in ethanolic KOH, pyruvic acid in NaOH, and/or diethyloxalate in THF/DMF to provide the compounds **5b to 8b**, respectively. The chemical structures of compounds **5b-8b** were confirmed by considering both spectral measurements and elemental analyses. In a similar way, under the same above-mentioned experimental conditions, the reaction of compound **1a,b** with 5-bromopyrimidin-2-amine **(2c)** takes place to give **3c,d** compounds respectively. The triazole-3,4-diamines **3c,d** were cyclized with each of diphenyl(choro-methyl)phosphanoxide **(4)**, phenacyl bromide, pyruvic acid, and diethyloxalate to give the corresponding compounds **5c,d–8c,d**, respectively, The chemical structures of compounds**5c,d–8c,d** were elucidated by their spectral measurements and elemental analyses data.

**Figure 2:**
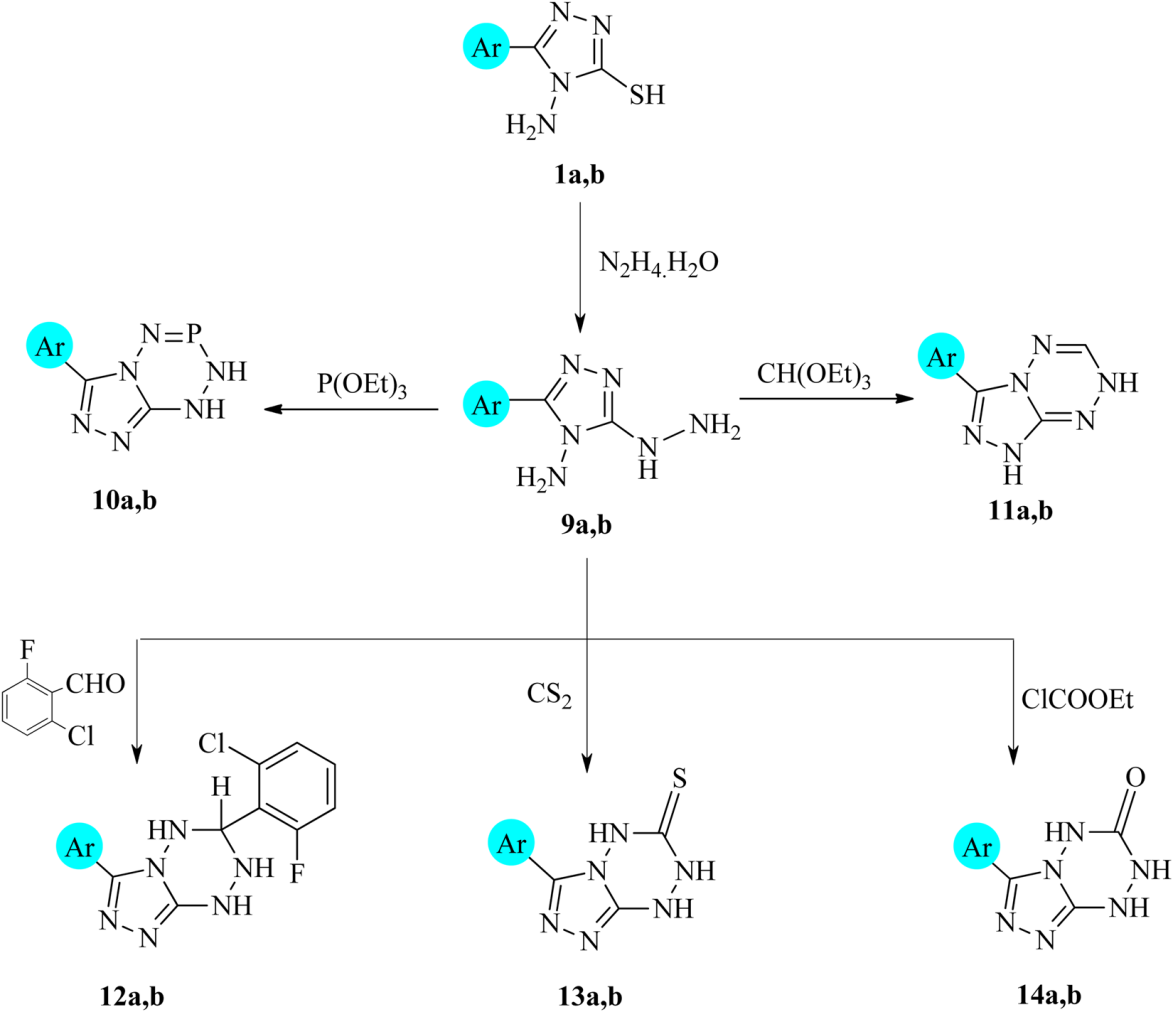
Synthesis of triazolotetrazine derivatives 1**0–14**.

The reported results of the high potency of 1,2,4-triazolo[1,2,4,5]tetrazines system as antimicrobial and anti-inflammatory agents were our motivation to synthesize several derivatives of these rings. Thus, it was found that refluxing compound **1a,b** with hydrazine hydrate dissolved in ethanol gives the corresponding 5 hydrazine-4-amino-1,2,4-triazoles (**9a,b**) which was used as new synthons for the present study aiming to build new 1,2,4-triazolotetrazines. A hydrazino group is a stronger nucleophilic group and more basic if it is compared to the amino group in both compounds **9a**,**b**, which enhances the cyclization firstly followed by the amino center (Fig. 2). The triazolo[4,3-*e*][1–5] tetrazaphosphinines (**10a,b**) were obtained from refluxing **9a**,**b** with triethylphosphite in tetrahydrofuran (THF), respectively. Treating compound **9a,b** with triethylorthoformate under the previously mentioned conditions, produced the corresponding 3-(pyridin-4-yl)- and/or 3-phenyl-1,7-dihydro-[1,2,4]triazolo[4,3-*b*][1,2,4,5]-tetrazine **11a** and/or **11b**, respectively. Cycloaddition reaction, in boiling ethanol with a few drops of piperidine, of compounds **9a,b** with 2-chloro–6-fluorobenzeldehyde furnished the corresponding triazolotetrazine derivatives **12 a,b**, respectively **(**Fig. 2).

Structures of compounds **10a,b, 11a,b** and **12a,b** were elucidated by examining the data of IR spectra. The IR absorption bands at ν (cm^−1^): 3123–2962, 3120–2965 of (broad NH-NH), 1650, 1643 of (P-NH), 1528, 1522 of (C=N) for **10a, b**, respectively. The IR absorption bands at ν (cm^-1^): 3184–3132, 3188–3129 of (NH, NH of tetrazine and triazole), 1521, 1519 (C=N) for **11a,b**, respectively. The IR absorption bands at ν (cm^−1^): 3354–3132, 3343–3129 (3NH), 3078, 3072 (aromatic (CH)), 1522, 1518 (C=N) for **12a,b**, respectively. The mass spectra of each of **10a** and **10b** gave molecular ion peaks at 218 and 219 which corresponded to the molecular weights of C_8_H_7_N_6_P and C_7_H_6_N_7_P of the assigned structures **10a**,**b**, respectively.

UV spectral data of both compounds **3a,b** and **12a,b** explains that the hetero-cyclization would inhibit the electronic transition and this caused the hypsochromic effect “shift to the shorter wavelength”. Thus, λ_max_ of **3a,b** was 372 and 374 nm while that of **12a,b** was 265 and 263 nm, respectively. ^13^C NMR (DMSO) of compound **12a** showed the presence of thirteen different signals for thirteen different carbon atoms, which, for **12b**, showed twelve signals for twelve different carbons. Finally, the compounds 1,2,4-trizolo[4,3 -b][1,2,4,5]tetrazine-5(4 *H*)-thiones/ones (**13a,b** and **14a,b**) were synthesized from refluxing of **9a,b** with CS_2_ in DMF and/or with ethyl chloroformate in THF/TEA, respectively (Fig. 2). Formation of **13a,b** occurs in two steps, the first, by addition to S=C=S and the second step is the elimination of H_2_S, while the formation of compounds **14a,b** was carried via esterification of **9a,b** followed by elimination of one molecule of ethanol in each case^52^. The chemical structures of **13a,b**, and **14a,b** were characterized by their elemental and spectral analyses. Thus, the infrared spectra of compounds **14a,b** showed absorption bands of NH, CONH at ν 3167, 3172 and 1643, 1651cm^−1^, respectively, while that of **13a,b** showed the absorption bands at ν 3332, 3325 cm^−1^ of NH and 1528, 1522 cm^−1^ of (C=S). The ^1^H NMR spectra of **13a,b** recorded signals of 3.8 (s, 1H, -NH- tetrazine at position 2), 5.3 (s, 1H, NH-tetrazine at position 4), 6.1 (s, 1H, NH-tetrazine at position 5) and 7.52–8.07 (m, 5H of benzene). ^13^C NMR gives us good evidence for the structure of **13a** where it showed resonated signals at 157 (C_3_ -triazole), 151 (C_5_ -triazole), 182 (C_3_ -tetrazine) and 130, 131,129, 127 ppm of benzene carbons. Mass spectra of both compounds **14a**,**14b** showed molecular ion peaks at 216 (4.7%) for **14a** and at 217 (6.2%) for **14b**, which confirmed their structures. In addition, ^13^C NMR data of **14a** and **14b** gave convincing evidence to their structures in which compound **14a** revealed the presence of seven different carbon signals, while **14b** showed six different carbon signals, which were in good agreement with the proposed structures (Fig. 2).

#### Biological activity

**Figure 3:**
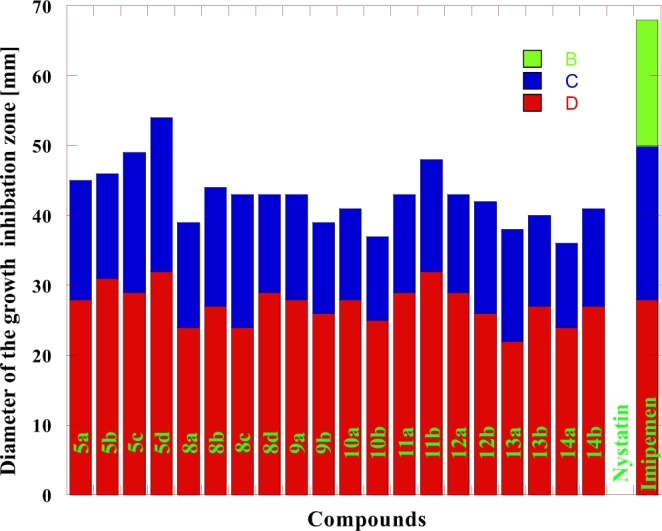
The antibacterial activity comparison of some tested compounds with Nalidixic acid and Imipenem toward *E. coli* at different concentrations where: (**A**) 25 µg/mL (**B**) 50 µg/mL (**C**) 100 µg/mL.

**Figure 4:**
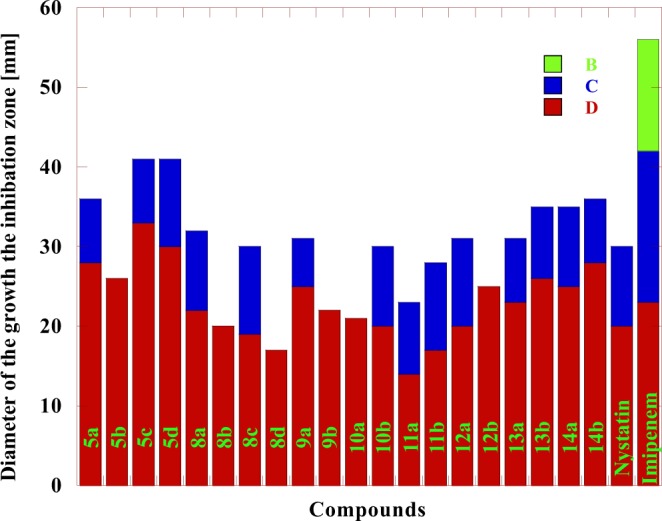
The antibacterial activity comparison of some tested compounds with Nalidixic acid and Imipenem toward *P.aeruginosa* at different concentrations where: (**A**) 25 µg/mL (**B**) 50 µg/mL (**C**) 100 µg/mL.

**Figure 5:**
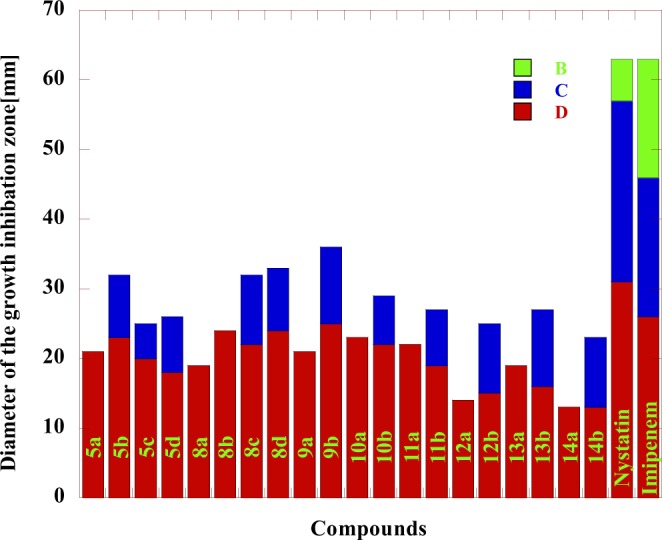
The antibacterial activity comparison of some tested compounds with Nalidixic acid and Imipenem toward *S. aureus* at different concentrations where: (**A**) 25 µg/mL (**B**) 50 µg/mL (**C**) 100 µg/mL.

**Figure 6:**
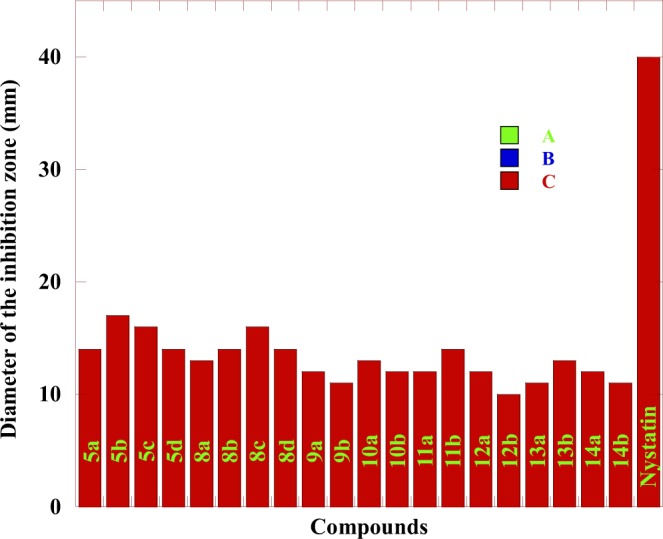
The antifungal activity comparison of some tested compounds with Nystatin toward *C. albicans* at different concentrations where: (**A**) 25 µg/mL (**B**) 50 µg/mL (**C**) 100 µg/mL.

The different biological activities of the synthesized compounds have been evaluated by studying their antibacterial activity against *Pseudomonas aeruginosa* and *Escherichia coli* as examples for Gram-negative bacteria *and Staphylococcus aureus* as an example for Gram-positive bacteria, in addition, the antifungal activity against *Candida albican*s using the technique reported by Barry *et al*.^53,54^. Dimethylformamide was used as a solvent. Nystatin was used as a reference drug for fungi while Nalidixic acid and Imipenem were used as reference drugs for bacteria. The diameter of the growth inhibition zone (DIZ) was presented in Tables 1, 2 and Figs. 3–6. The Minimum Inhibitory Concentrations (MIC) in antibacterial activity were presented in Table 1.

**Table 1:** The antibacterial activity for some of the newly prepared Compounds.

*S. aureus*				*P.aeruginosa*				*E. coli*				Bacteria
MIC	25	50	100	MIC	25	50	100	MIC	25	50	100	Inhibitory Concentrations
Growth inhibition Zone (mm)^*^												Compounds
75.7	—	—	21	49.6	—	8	28	38.6	—	17	28	5a
48.5	—	9	23	68	—	—	26	44.3	—	15	31	5b
49.6	—	5	20	49.7	—	8	33	32	—	20	29	5c
48.9	—	8	18	46.9	—	11	30	31.7	—	22	32	5d
68.2	—	—	19	47.9	—	10	22	38.3	—	15	24	8a
67.2	—	—	24	79.6	—	—	20	37.3	—	17	27	8b
46.9	—	10	22	43.3	—	11	19	26.6	—	19	24	8c
48.4	—	9	24	79.5	—	—	17	44.6	—	14	29	8d
68.7	—	—	21	49	—	6	25	42.6	—	15	28	9a
46.6	—	11	25	61.3	—	—	22	44.5	—	13	26	9b
75.1	—	—	23	75.4	—	—	21	45.5	—	13	28	10a
49.5	—	7	22	45	—	10	20	45.7	—	12	25	10b
66.5	—	—	22	43.8	—	9	14	25.7	—	14	29	11a
48.2	—	8	19	40.4	—	11	17	43.8	—	16	32	11b
75.3	—	—	14	44.4	—	11	20	44.6	—	14	29	12a
39.5	—	10	15	53.5	—	—	25	38.7	—	16	26	12b
61.7	—	—	19	49.3	—	8	23	30.3	—	16	22	13a
37.2	—	11	16	49	—	9	26	45.2	—	13	27	13b
73.6	—	—	13	47.7	—	10	25	44.5	—	12	24	14a
31.6	—	10	13	49.6	—	8	28	43.7	—	14	27	14b
23.3	6	26	31	45.3	—	10	20	—	—	—	—	Nalidixic acid
14.2	17	20	26	15.9	14	19	23	13.9	18	22	28	Imipenem

**Table 2:** The antifungal activity for some of the newly prepared Compounds.

*C. albicans*			Fungi
25	50	100	Inhibitory Concentrations
Growth inhibition Zone (mm)^*^			Compounds
—	—	14	5a
—	—	17	5b
—	—	16	5c
—	—	14	5d
—	—	13	8a
—	—	14	8b
—	—	16	8c
—	—	14	8d
—	—	12	9a
—	—	11	9b
—	—	13	10a
—	—	12	10b
—	—	12	11a
—	—	14	11b
—	—	12	12a
—	—	10	12b
—	—	11	13a
—	—	13	13b
—	—	12	14a
—	—	11	14b
—	—	40	Nystatin

• Synthesized compounds (Inhibitor) Concentrations: 100, 50 and 25 μg /mL.

Highly active: DIZ ≥ 12.

Moderately active: DIZ = 9–12.

Slightly active: DIZ = 6–9;

Not sensitive: DIZ < 6 mm.

The results in Table 1 show that the antibacterial activity of the tested compounds could be classified to higher to moderate activity against the used bacteria *E.coli, S. aureus and P. aeruginosa* in comparison with Nalidixic acid at concentrations 100, 50 and 25 µg/mL. All the tested Compounds showed higher activity against *E. coli* bacteria at 100 µg/mL concentrations and higher to moderate activity at 50 µg/mL concentrations. For *S. aureus* bacteria, all the tested Compounds showed higher activity at 100 µg/mL concentrations. Compounds **5a, 5c, 8a, 8b, 9a, 10a, 11a, 12a, 13a** and **14a** were not sensitive toward *S. aureus* while compounds **5d, 10b** and **11b** were slightly active at 50 µg/mL concentrations. On the other hand, the remaining compounds showed moderate activity at 50 µg/mL concentrations. For *P. aeruginosa* bacteria, all the tested compounds showed higher activity at 100 µg/mL concentrations. Compounds **5b, 8b, 8d, 9b, 10a** and **12b** were not sensitive toward *P. aeruginosa* bacteria while compounds **5a, 5c, 9a, 13a** and **14b** were slightly active at 50 µg/mL concentrations. On the other hand, the remaining compounds showed moderate activity at 50 µg/mL concentrations. The MICs for all the tested compounds against *E.coli, S. aureus, and P. aeruginosa* were presented in Table 1. The data in Table 2 showed moderate to high antifungal activities of the tested compounds against *C. albicans* in comparison with Nystatin at 100 µg/mL concentration The MIC for all the tested compounds against *C. albicans* fungi was 100 µg/mL. The higher activity of some of the tested compounds was mainly due to containing chlorine, fluorine, bromine and phosphorus elements within the chemical structure of 1,2,4-triazine and 1,2,4,5-tetrazine^55^. On the other hand, the obtained results in Table 3 indicated that: Compounds **10a,b, 12a,b** and **13b** carrying phenyl and pyridine groups having both the chlorine and fluorine elements had good anti-inflammatory activity, in comparison with the standard anti-inflammatory drug used (Indomethacin). Compounds **10a,b**, and **12a,b** contained mainly triazole and tetrazine rings with the presence of both fluorine and phosphorus elements, incorporated with pyridyl moiety. The activity of the new materials depends on the existence of those new moieties which have a high biological activity^55^. The higher biological activity of the synthesized compounds was in a good agreement with the previously stated results in the field of fluorine and phosphorus-bearing nitrogen heterocyclic systems^9,56^.

**Table 3:** The anti-inflammatory activity for some of the newly synthesized compounds.

Compound	Dose (mg /Kg)	Paw edema(g)* ± S.E.	% inhibition
9a	25 5	0.57 ± 0.05 0.61 ± 0.06	13.63 7.57
9b	25 5	0.52 ± 0.05 0.59 ± 0.06	21.21 10.60
10a	25 5	0.42 ± 0.05 0.48 ± 0.06	36.36 27.27
10b	25 5	0.45 ± 0.05 0.49 ± 0.06	31.81 25.75
12a	25 5	0.41 ± 0.03 0.48 ± 0.06	37.87 27.27
12b	25 5	0.43 ± 0.05 0.47 ± 0.06	34.84 28.78
13a	25 5	0.55 ± 0.05 0.64 ± 0.05	16.66 3.03
13b	25 5	0.40 ± 0.05 0.46 ± 0.06	39.39 30.30
Control	0	0.66 ± 0.05	0
Indomethacin	5	0.32 ± 0.02	51.51

^*^Significant difference from the control value at p < 0.05.

##### Molecular modeling studies (Structure-based drug design)

**Figure 7:**
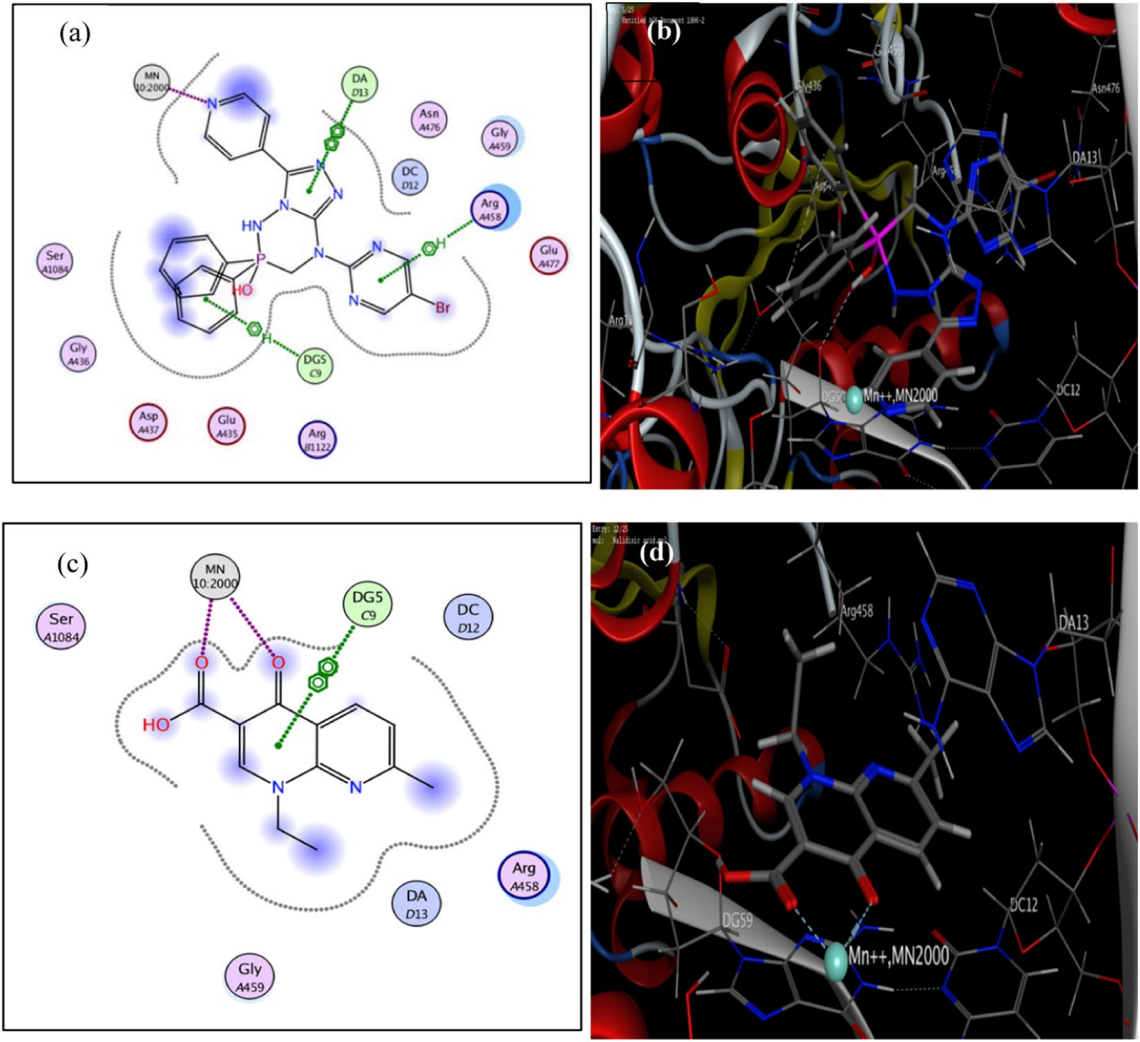
Molecular docked model of compound **5d** and **Nalidixic acid** with DNA Gyrase enzyme (the target is presented as thin sticks; the ligands are drawn as ball-and-stick). Images (**a,c**) represent the 2D docking styles for DNA Gyrase enzyme with compound **5d** and **Nalidixic acid**, respectively. Images (**b,d**) represent the 3D docking styles for compound **5d** and **Nalidixic acid**, respectively.

 Table 4 and Fig. 7 illustrate the results of the bonding interactions for the docking of compound **5d** and **Nalidixic acid** with amino acids of DNA Gyrase enzyme (PDB ID: 2XCT) active site. From these results, we found that the active compound **5d** showed extra binding modes to DA13 and Arg 458 in addition to interaction with the essential binding sites Mn metal and DG5.

**Table 4:** Molecular modeling results for compound 5d and Nalidixic acid during docking in DNA Gyrase enzyme (PDB ID: 2XCT) active site.

Compound	COX-2 (PDB: 2XCT)					
	Affinity Kcal/mol	Distance (in Å) from main residue		Functional group	Interaction	2d caption(3d caption)
**5d**	−14.2589	1.62 3.95 4.76 3.93 3.73	Mn Arg458 DG5 DA13 DA13	Pyridine N Diazine ring Phenyl ring Triazole ring Triazole ring	Metal Bond pi-H pi-H pi-pi pi-pi	Fig. 7a (Fig. 7b)
Nalidixic acid	−10.0699	1.71 3.99 3.67	Mn DG5 DG5	Carbonyl group Phenyl ring Phenyl ring	Metal Bond pi-pi pi-pi	Fig. 7c (Fig. 7d)

**Figure 8:**
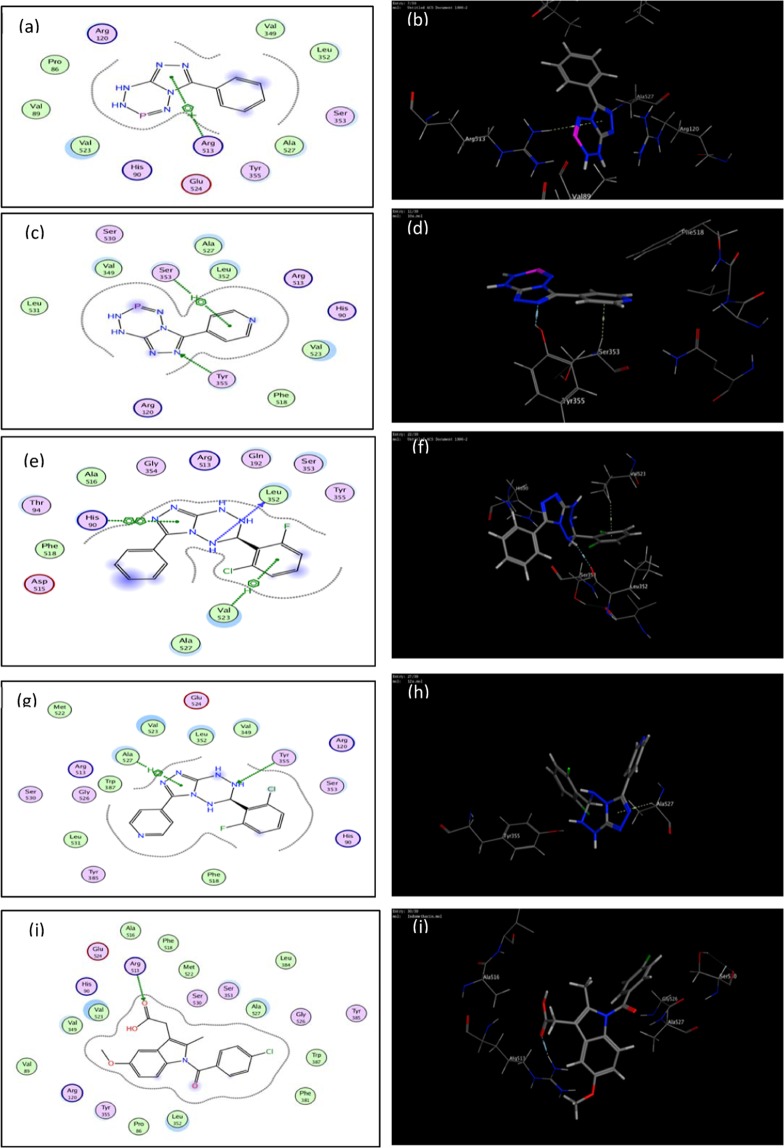
Molecular docked model of compounds **10a,b, 12a,b** and Indomethacin with COX–II enzyme (the target is presented as thin sticks; the ligands are drawn as ball-and-stick). Images (**a,c,e,g,i**) represent the 2D docking styles for COX–II enzyme with compounds **10a,b, 12a,b** and Indomethacin, respectively. Images (**b,d,f,h,j**) represent the 3D docking styles for COX–II enzyme with compounds **10a,b, 12a,b** and Indomethacin, respectively.

Table 5 and Fig. 8 illustrate the results and the bonding interactions of the docked compounds and **Indomethacin**, respectively, with active sites of COX-2 (PDB ID: 1CX2).

**Table 5:** Molecular modeling results for compounds and Indomethacin during docking in COX-2 (PDB ID: 1CX2) active site.

Compound	COX-2 (PDB: 1CX2)					
	Affinity Kcal/mol	Distance (in Å) from main residue		Functional group	Interaction	2d caption (3d caption)
10a	−17.8710	4.47	Arg513	Triazole ring	pi-cation	Fig. 8a (Fig. 8b)
10b	−16.1990	2.47 3.71	Tyr355 Ser353	Triazole N Pyridine ring	H-acceptor pi-H	Fig. 8c (Fig. 8d)
12a	−15.3810	3.23 3.80 3.56	Leu352 Val523His90	-NH- Phenyl ring Triazole ring	H-donor pi-H pi-pi	Fig. 8e (Fig. 8f)
12b	−16.2058	3.86 3.37	Tyr355 Ala527	-NH- Triazole ring	H-acceptor pi-H	Fig. 8g (Fig. 8h)
Indomethacin	−15.8796	2.70	Arg513	Carbonyl	H-acceptor	Fig. 8i (Fig. 8j)

From these results, it appears that, generally, the tested compounds and Indomethacin showed a comparable binding pattern. Compound **10a** showed well interaction with Arg513 residue. Compound **10b** showed well interaction with Ser353 and Tyr355 which is going alongside with the screening results. Also, compound **12a** showed three binding interactions to Leu352, His90 and Val523 amino acid. Finally, compound **12b** binds to Ala527 and Tyr355 residues.

## Conclusions

Some novel heterocyclic compounds **5a,b**–**14a,b** containing fluorine, chlorine, bromine and phosphorus elements were synthesized utilizing 3-substituted-4-amino-5-substituted amino-1,2,4-triazoles **3a-d** and 3-substituted-4-amino-5-hydrazino-1,2,4-triazole **9a,b** compounds as building units in the synthesizing process. These newly prepared compounds were fully characterized through the spectral and elemental analyses which were completely fit with the assigned structures. A number of the synthesized compounds were screened against gram-positive bacteria, gram-negative bacteria, and fungi, such as *Streptococcus aureus, Escherichia coli, Pseudomonas aeruginosa*, and *Candida albicans*. Most of the newly prepared compounds showed a high antibacterial, antifungal, and anti-inflammatory in comparing with the standard commercial antibiotics Imipenem and Nalidixic acid, Nystatin and Indomethacin, respectively. Compounds **10a,b**, and **12a,b** containing both chlorine and fluorine elements showed high inflammation activity.
